# Altered Microstate Dynamics and Spatial Complexity in Late-Life Schizophrenia

**DOI:** 10.3389/fpsyt.2022.907802

**Published:** 2022-06-27

**Authors:** Gaohong Lin, Zhangying Wu, Ben Chen, Min Zhang, Qiang Wang, Meiling Liu, Si Zhang, Mingfeng Yang, Yuping Ning, Xiaomei Zhong

**Affiliations:** ^1^Center for Geriatric Neuroscience, The Affiliated Brain Hospital of Guangzhou Medical University, Guangzhou, China; ^2^The First School of Clinical Medicine, Southern Medical University, Guangzhou, China; ^3^Guangdong Engineering Technology Research Center for Translational Medicine of Mental Disorders, Guangzhou, China

**Keywords:** late-life schizophrenia, microstate, omega complexity, spatial complexity, electroencephalogram

## Abstract

**Background:**

Resting-state EEG microstate and omega complexity analyses have been widely used to explore deviant brain function in various neuropsychiatric disorders. This study aimed to investigate the features of microstate dynamics and spatial complexity in patients with late-life schizophrenia (LLS).

**Method:**

Microstate and omega complexity analyses were performed on resting-state EEG data from 39 in patients with LLS and compared with 40 elderly normal controls (NCs).

**Result:**

The duration of microstate classes A and D were significantly higher in patients with LLS compared with NCs. The occurrence of microstate classes A, B, and C was significantly lower in patients with LLS compared with NCs. LLS patients have a lower time coverage of microstate class A and a higher time coverage of class D than NCs. Transition probabilities from microstate class A to B and from class A to C were significantly lower in patients with LLS compared with NCs. Transition probabilities between microstate class B and D were significantly higher in patients with LLS compared with NCs. Global omega complexity and anterior omega complexity were significantly higher in patients with LLS compared with NCs.

**Conclusion:**

This study revealed an altered pattern of microstate dynamics and omega complexity in patients with LLS. This may reflect the disturbed neural basis underlying LLS and enhance the understanding of the pathophysiology of LLS.

## Introduction

Late-life schizophrenia (LLS) is a severe mental disorder in older people, seriously impairing the daily functioning and quality of life of LLS patients. With the intensification of global population aging, schizophrenia in late life has become a major public health issue worldwide (1). LLS differs from schizophrenia in younger individuals in many respects. Older patients with schizophrenia have fewer severe positive symptoms and more severe negative symptoms than younger patients (2, 3). Moreover, patients with LLS have more serious cognitive impairment (4, 5), complicated medical comorbidity (6, 7), and changed brain structure and function due to aging (8, 9) compared with younger patients. Currently, most studies on schizophrenia are conducted on the young to middle-aged population. Research on older patients with schizophrenia is relatively scarce (10).

The pathophysiology of LLS remains elusive. Accumulating evidence suggests that disturbed brain networks may underlie the symptomatology of schizophrenia (11–13). The process of brain networks is highly dynamic, functioning in a millisecond timescale. EEG microstate analysis, with a high temporal resolution, can be used to investigate the dynamic properties of large-scale brain networks. EEG microstates represent the spatial topography of scalp electric potential that remains quasi-stable for a short period and then changes to a different topography (14). EEG microstates occur in a sub-second duration of 60–120 ms, compatible with the time range of information operations (15). Four canonical microstates labeled as classes A, B, C, and D have been consistently identified in previous studies. One hypothesis proposed that an EEG microstate represents certain neural assemblies, and transitions between microstate classes reflect sequential activation of different brain networks (16). Growing evidence has shown that resting-state EEG microstates are closely related to resting-state networks (17–20). Simultaneous EEG-fMRI found a correlation between resting-state networks and each class of the four microstates: class A (auditory network), class B (visual network), class C (saliency network), and class D (attention network). Altered patterns of EEG microstates may reflect disturbed brain networks and have been reported in several neuropsychiatric disorders, such as Lewy body dementia (21), Alzheimer's disease (22), and bipolar disorder (23).

A growing body of research has found altered properties of EEG microstates in patients with schizophrenia (24–26). Such findings were interpreted as reflecting the brain network malfunction underlying the symptomatology of schizophrenia. In addition, the same deviant microstate abnormalities as in schizophrenia patients were found in high-risk individuals and unaffected relatives, indicating that EEG microstates may serve as an endophenotype for schizophrenia (27, 28). Moreover, links have been found between abnormal microstate patterns and schizophrenic symptoms (29, 30). In terms of clinical translation, microstate-based neurofeedback training has proven to be a candidate treatment for schizophrenia (31). Microstate was found to be an effective indicator of symptom improvements in TMS therapy for schizophrenia (32). Furthermore, microstate-based machine learning was tested and shown to be capable of effectively distinguishing those with schizophrenia from healthy people (33, 34).

Like EEG microstates, omega complexity can be used to measure the functioning of large-scale brain networks. Omega complexity is a linear indicator for spatial complexity extracted from the covariance matrix based on multiple channel EEG data (35). It can apply to the whole brain or to a specific brain area. Omega complexity assesses the degree of synchronization or coordination between spatially distributed neural processes. Therefore, it can reflect the connectivity of the neural network. Several studies revealed altered omega complexity in different cognitive processes (36, 37) and showed abnormality in various neuropsychiatric diseases (38, 39). Increased omega complexity has been found in schizophrenic patients, reflecting loosened brain networks in schizophrenia (40–42).

EEG microstate and omega complexity analyses have revealed brain network disturbance in the temporal and spatial domains in schizophrenia and other disorders. However, as mentioned before, the underlying pathophysiology of LLS may differ from that found in schizophrenia in younger people. Therefore, it is necessary to conduct studies specifically for LLS. This study aims to investigate the disturbance of brain networks in LLS through resting-state EEG microstate analysis and omega complexity analysis. Based on the above evidence, it was hypothesized that microstate dynamics and omega complexity are altered in LLS compared to those in healthy older people. Our findings provide a deeper understanding of the pathophysiology of LLS and new insights into the treatment of LLS patients.

## Materials and Methods

### Participants

EEG data were collected from 39 inpatients with LLS and 40 older adult normal controls (NCs). Inpatients with LLS were from the Department of Geriatric Psychiatry in the Affiliated Brain Hospital of Guangzhou Medical University. All patients met the DSM-IV criteria for schizophrenia. The diagnosis was confirmed by two independent, experienced psychiatrists. All patients were over 60 years of age. The exclusion criteria included a history of neurological diseases such as brain tumors, Parkinson's disease, stroke, severe head injury, and alcohol or drug abuse. All patients were receiving neuroleptic medication. Gender and age-matched NCs were recruited from the local communities. All NCs were screened for psychiatric disorders using the Mini International Neuropsychiatric Interview, 4th Edition (MINI). All NCs reported an absence of a family history of psychosis.

### EEG Recording and Preprocessing

Resting-state EEG data were recorded by a 21-channel Nicolet One System (Natus^®^, Germany) at a sampling rate of 125 Hz with a bilateral mastoids reference for inpatients with LLS and a 64-channel Neuroscan quick-cap (Neuroscan Labs, USA) at a sampling rate of 1000 Hz with a nasal reference for NCs. EEG recording electrodes were placed according to the international 10–20 system with auxiliary electrodes for artifact detection. All electrode impedance was maintained below 5 kΩ during EEG recording. Patients were asked to sit comfortably with their eyes closed in a dimly lit room during the recording.

Resting-state EEG data were imported to MATLAB (Mathworks, v2013a) for preprocessing using the EEGLAB toolbox (43). EEG data were transformed into a 19-channel montage (FP1, FP2, F3, F4, Fz, F7, F8, C3, C4, Cz, P3, P4, Pz, O1, O2, T3, T4, T5, and T6) and were resampled to 125 Hz. EEG data were band-pass filtered between 1 and 60 Hz, and a notch filter was used to remove power line interference. Afterward, continuous data were segmented into 2,000 ms epochs. Visual inspection was used to eliminate the epochs contaminated by severe noise. Bad channels were removed and interpolated using spherical spline interpolation. Independent component analysis was performed to remove electrooculography, electromyography, and any other non-physiological artifacts. Then EEG data were recomputed against an average reference. The first 20 artifact-free epochs were selected for further analysis. There was no significant difference in the number of bad epochs rejected between groups (*t* = 0.177, *p* = 0.860). See the Supplementary Material for a flowchart of the process of EEG preprocessing.

### EEG Microstate Analysis

**Figure 1:**
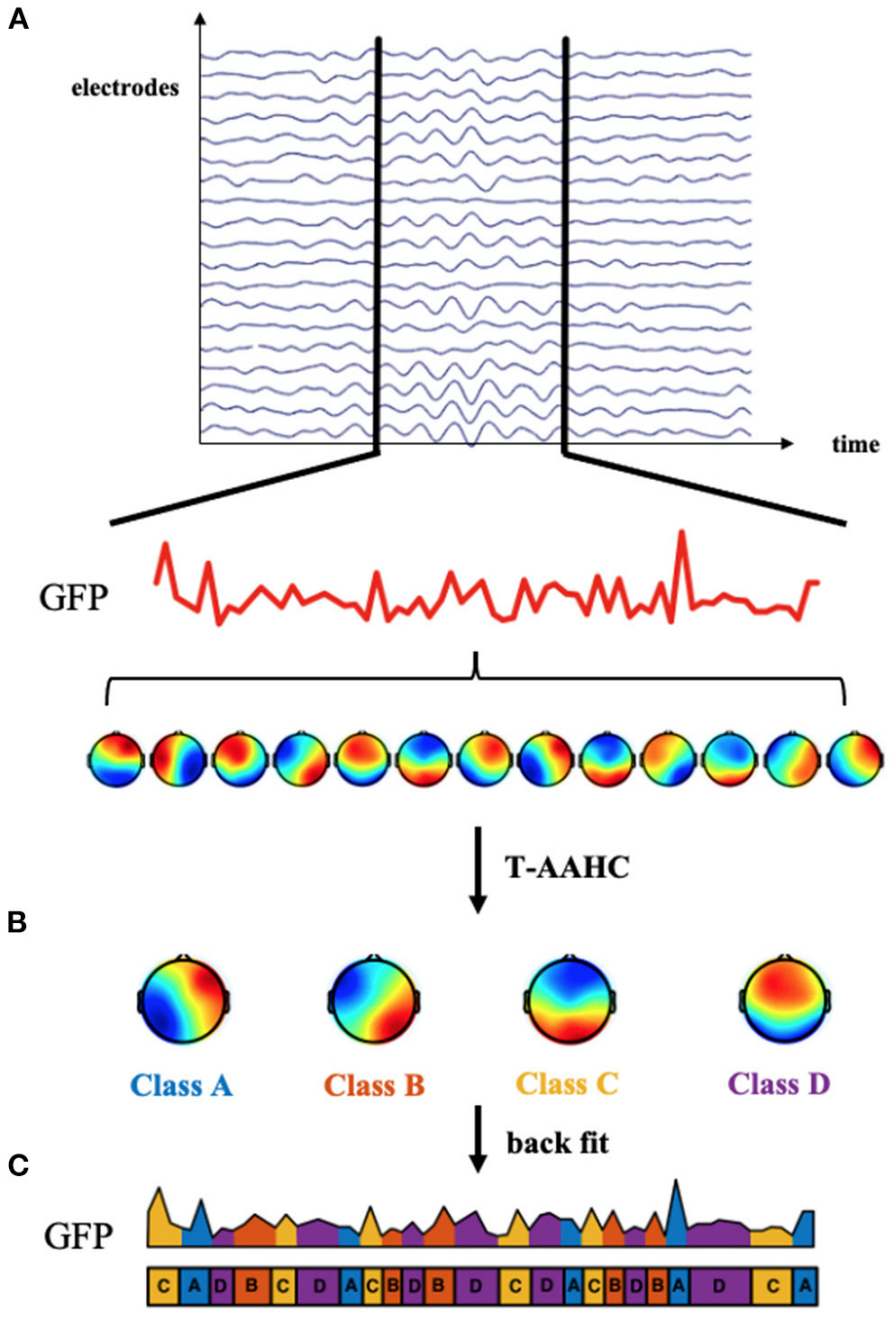
Microstate analysis methods. **(A)** The global field power (GFP) was computed at each time point. The topographies of electric potential at peaks of the GFP curve were extracted because GFP peaks have the highest signal-to-noise ratio. **(B)** These topographies were submitted to the Topographic Atomize and Agglomerate Hierarchical Clustering Algorithm (T-AAHC). Four canonical classes labeled **(A–D)** were assigned to each cluster. **(C)** Four microstate classes were back-fitted to continuous EEG data based on the criterion of maximal spatial correlation. Then, the microstate parameters and syntax could be calculated to quantify the microstate dynamics.

EEG microstate analysis was performed using the EEGLAB toolbox and custom scripts based on MATLAB. First, EEG data were band-pass filtered between 2 and 20 Hz. The global field power (GFP) for each time point was computed, representing the variance of potential across all electrodes at a certain instance. The scalp topographies at peaks of GFP were extracted since these topographies have the highest signal-to-noise ratio and stability. Topographies for each group were submitted to the Topographic Atomize and Agglomerate Hierarchical Clustering Algorithm (T-AAHC). The number of clusters was set as four for better comparability with early studies (24, 44). For each group, four prototype microstate maps were yielded and then back-fitted to EEG data based on the criterion of maximal spatial correlation. These four maps were labeled as classes A, B, C, and D according to previous research based on a well-established standard (44), whereby class A exhibits a right frontal to left occipital orientation, class B exhibits a left frontal to right occipital orientation, class C has a prefrontal to occipital orientation, and class D shows a frontocentral to occipital orientation. Three microstate parameters (duration, occurrence, and time coverage) and microstate syntax were computed to quantify the temporal features of EEG microstates. Duration refers to the mean time coverage of a given microstate class. The occurrence is the number of times a certain microstate class occurs per second. The time coverage is the percentage of total occupied time for a given microstate class. Microstate syntax refers to transition probabilities from one class to another. See Figure 1 for an overview of the microstate analysis pipeline.

### Spatial Complexity

Omega complexities were computed using MATLAB custom scripts in the following procedures: (1) For global omega complexity, all 19 EEG data electrodes were used to construct a 19×19 matrix. For anterior omega complexity, the data from electrodes FP1, FP2, F3, F4, Fz, F7, and F8 were selected to construct a 7×7 matrix. For posterior omega complexity, the data from electrodes O1, O2, P3, P4, Pz, T5, and T6 were selected to construct a 7×7 matrix. (2) Principal component analysis was used to compute the eigenvalues (λ) of each matrix. Eigenvalues represent the contribution of each component to the total variance. (3) To evaluate the relative contribution of each component, the eigenvalues were normalized to unit sum using the following equation:


(1) $$\begin{array}{c} \lambda_{i}^{{}^{\prime }}=\frac{\lambda_{i}}{\sum_{i=1}^{k}\lambda_{i}} \end{array}$$

Where, *i* refers to the number of the electrode, and λ' refers to the normalized eigenvalue. (4) The value of omega complexity (Ω) was calculated with the following equation:


(2) $$\Omega =\text{exp}\left\{-\;\sum_{i=1}^{k}\lambda_{i}^{{}^{\prime }}log\lambda_{i}^{{}^{\prime }}\right\}$$

The values of omega complexity vary from 1 to k (the total number of electrodes used), in which 1 represents the minimal spatial complexity and maximal synchronization, and vice versa. See Supplementary Material for a flowchart of omega complexity analysis.

### Statistics

Statistical analyses were performed using SPSS 26 (IBM, USA). The significance level was set at 0.05. All statistical tests were two-tailed. For demographic characteristics, the independent student's *t*-test and chi-square test were used to compare continuous variable and categorical variables between groups, respectively. For each microstate parameter, a repeated-measures ANCOVA was performed with microstate classes (A–D) as a within-subject factor, group (LLS, NCs) as a between-subject factor, and age as a covariate. In cases where the interaction effect between microstate class and group was significant, a *post hoc* univariate ANCOVA was performed using the same covariate mentioned above. A univariate ANCOVA was performed with age as a covariate for each pair of transition probabilities. A Bonferroni correction was used for multiple comparisons. A univariate ANCOVA was performed with age as a covariate for global and regional omega complexity.

## Results

### Demographic and Clinical Characteristics

Table 1 shows the demographic and clinical characteristics. There was no significant difference between groups.

**Table 1:** Demographic and clinical characteristics of late-life schizophrenia patients (LLS) and normal controls (NCs).

	**LLS**	**NC**	**t/χ^2^**	***p***
Gender (F/M)	29/10	29/11	0.035	0.852
Age (years)	68.23 ± 5.43	69.30 ± 6.93	0.762	0.449
Education (years)	9.30 ± 2.65	10.21 ± 3.26	1.350	0.181
Disease duration (years)	34.20 ± 13.38			
Admission due to schizophrenia (times)	4.13 ± 4.52			
Antipsychotic dose (mg/day)	208.87 ± 130.03			

*Values of continuous variables are shown as mean ± standard deviation. The antipsychotic dose was measured by chlorpromazine equivalents*.

### Microstate Analysis

**Figure 2:**
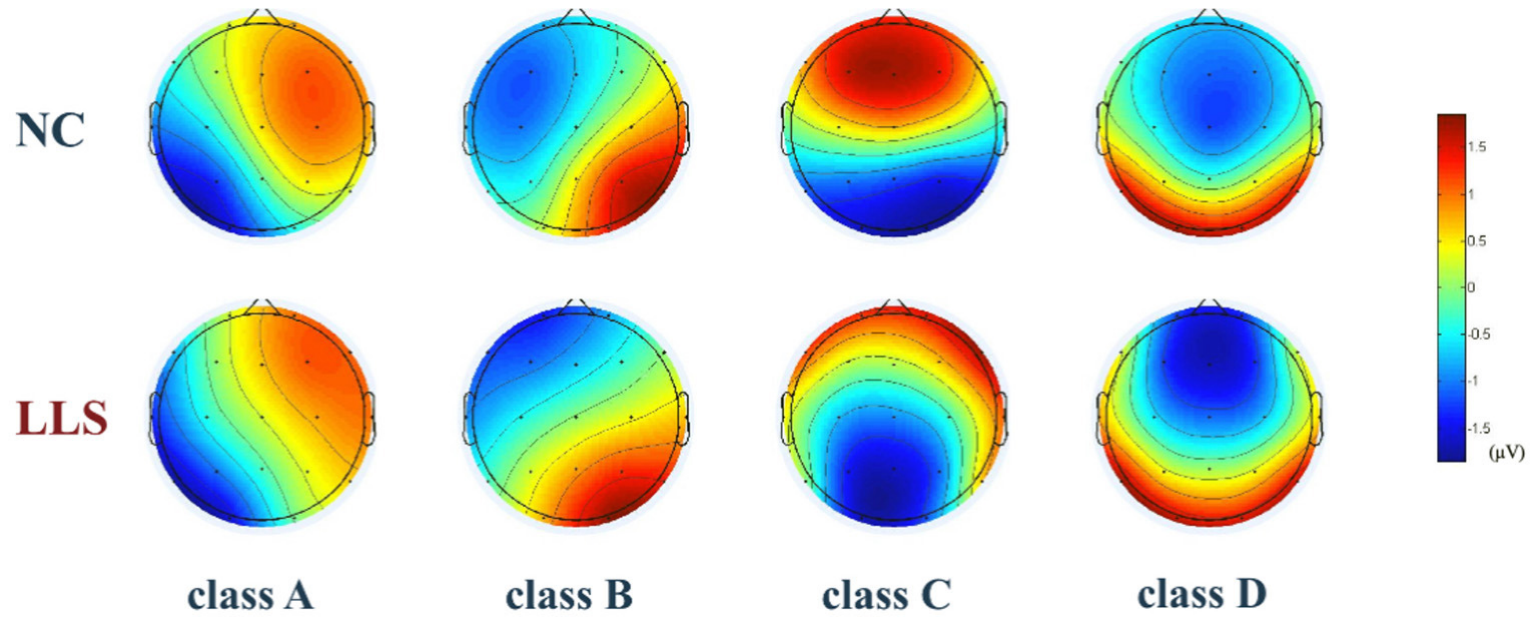
The topographical maps of the four microstate classes (A–D) in the late-life schizophrenia group (LLS) and normal controls group (NCs). Classes A to D were labeled according to previous literature based on a well-established descriptive standard, whereby class A exhibits a right frontal to left occipital orientation, class B exhibits a left frontal to right occipital orientation, class C has a prefrontal to occipital orientation, and class D shows a frontocentral to occipital orientation. Color is used to represent electric potential. Red represents positive values, and blue represents negative values (the polarity in microstate can be inverted).

Figure 2 shows the topographical maps of the four microstate classes for patients with LLS and for NCs. Repeated-measures ANCOVAs yielded significant main effects for the groups in duration (*F* = 11.833, *p* < 0.001, η2 = 0.135) and occurrence (*F* = 21.332, *p* < 0.001, η2 = 0.219), revealing an increased mean duration and a decreased mean occurrence in patients with LLS compared with NCs. There is also a significant microstate class × Group interaction for duration (*F* = 3.040, *p* = 0.034, η2 = 0.110), occurrence (*F* = 6.321, *p* < 0.001, η2 = 0.204) and time coverage (*F* = 3.794, *p* = 0.014, η2 = 0.133). *Post hoc* one-way ANCOVAs showed that patients with LLS had significantly increased duration (*F* = 6.656, *p* = 0.012, η2 = 0.081), decreased occurrence (*F* = 30.884, *p* < 0.001, η2 = 0.289), and decreased time coverage (*F* = 5.031, *p* = 0.028, η2 = 0.062) of microstate class A compared with NCs. There was also a significant increase in duration (*F* = 22.589, *p* < 0.001, η2 = 0.229) and time coverage (*F* = 8.489, *p* = 0.005, η2 = 0.100) of microstate class D for patients with LLS compared with NCs. For class B (*F* = 9.873, *p* = 0.002, η2 = 0.115) and class C (*F* = 4.217, *p* = 0.043, η2 = 0.053), patients with LLS had significantly decreased occurrence compared with NCs.

**Figure 3:**
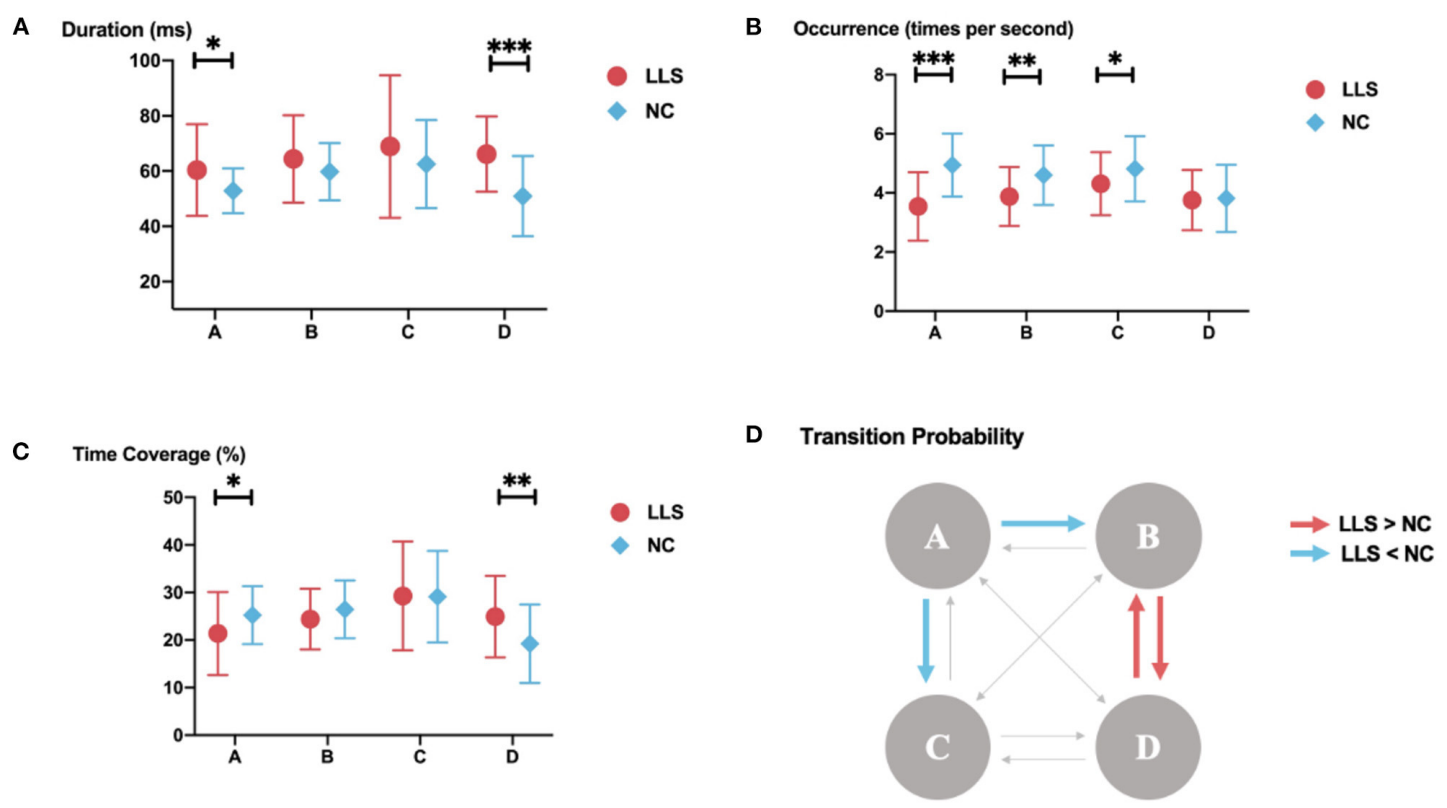
Results of the microstate analysis for late-life schizophrenia (LLS) and normal controls (NCs). **(A–C)** microstate parameters for late-life schizophrenia and normal controls. **(A)** duration; **(B)** occurrence; **(C)** time coverage for late-life schizophrenia and normal controls. The parameters of each class are displayed from left to right. Red icons indicate parameters of late-life schizophrenia, blue icons indicate parameters of normal controls, and lines through icons indicate standard deviation. ^*^indicates *p-*value ≤ 0.05, ^**^indicates *p-*value ≤ 0.01, ^***^indicates *p-*value ≤ 0.001. **(D)** results of syntax analysis. Red arrows indicate significantly higher transition probabilities for patients with late-life schizophrenia compared with to controls. Blue arrows indicate significantly lower transition probabilities for patients with late-life schizophrenia compared with to controls.

For microstate syntax analysis, we found significantly decreased transition probabilities from classes A to B (*F* = 9.641, *p* = 0.032, η2 = 0.113) and from classes A to C (*F* = 9.696, *p* = 0.031, η2 =0.113) in patients with LLS compared with NCs. Transition probabilities were also found to significantly increase from classes B to D (*F* = 10.111, *p* = 0.026, η2 = 0.117) and from classes D to B (*F* = 10.675, *p* = 0.020, η2 = 0.123) in patients with LLC compared with NCs. Figure 3 shows the details.

### Omega Complexity

**Figure 4:**
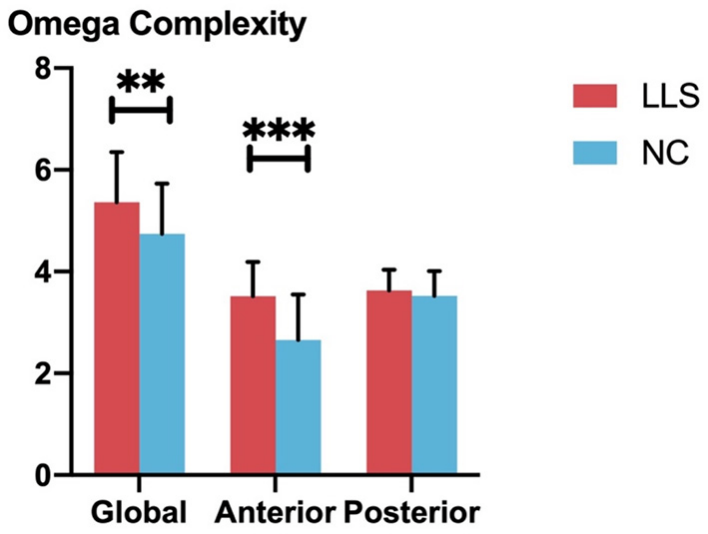
Comparison of global and regional omega complexity between patients with late-life schizophrenia (LLS) and normal controls (NCs). Red bars indicate the omega complexities of patients with LLS, and blue bars indicate the omega complexity of NCs. From left to right, three pairs of bars indicate global omega complexity, anterior complexity, and posterior complexity for each group, respectively. Standard deviations are indicated using the error bar. ^**^indicates *p-*value ≤ 0.01, ^***^indicates *p*-value ≤ 0.001.

Univariate ANCOVAs revealed that global omega complexity is significantly higher in patients with LLS compared with NCs (*F* = 7.274, *p* = 0.009, η^2^ = 0.087). For regional omega complexity, we found that anterior omega complexity is significantly higher for patients with LLS compared with NCs (*F* = 22.819, *p* < 0.001, η^2^ = 0.231). However, there is no significant difference in posterior omega complexity between groups. Figure 4 shows the details.

## Discussion

In the current study, EEG microstate and omega complexity analyses were used to investigate the pathophysiology of patients with LLS. Microstate analysis revealed altered parameters for certain microstate classes in patients with LLS. An altered pattern of microstate syntax was also found. Omega analysis revealed that patients with LLS have higher global complexity and higher anterior complexity.

A large body of prior literature found increased parameters of class C and decreased parameters of class D in patients with schizophrenia compared with healthy controls (15, 27, 28). These findings show a certain consistency. A meta-analysis including seven studies reported that microstate class C was more frequent and microstate class D was shorter in schizophrenia than in controls with a medium effect size (24). Simultaneous EEG-fMRI recording found that microstate class C was associated with the salience network (17), which functions to identify the most relevant stimulus from enormous internal and external inputs to guide appropriate actions (45). Therefore, altered parameters of class C in schizophrenia may reflect a disturbance in the salience network, in line with the hypothesis that a disarranged salience network underlies the difficulty of schizophrenic patients in differentiating between the inner world and the outside world (46). Similarly, microstate class D is related to the frontoparietal attention network (17). Deviant dynamics of class D in schizophrenia may reflect impaired cognitive functions involving attentional processes, such as have been widely found in patients with schizophrenia (47). However, this consistent pattern of schizophrenia is contrary to our findings.

We found increased duration and time coverage of class D and decreased occurrence of class C in patients with LLS compared with NCs. The reasons that may account for the discrepancy are as follows. Firstly, despite the opposite direction, our findings of deviant parameters of classes C and D also suggest disorganized salience networks and attention networks in patients with LLS. The opposite direction of our findings compared to those reported by previous studies may be explained by the differences between older and younger patients with schizophrenia in terms of pathophysiology and symptomology. A possible explanation is that patients with LLS have less prominent positive symptoms, which correlate negatively with the duration of class D (31, 48). Aging-related changes in brain function may also partially account for this difference. However, the small number of studies on LLS makes further interpretation challenging. In addition, the heterogeneity of patient characteristics between studies may contribute to inconsistencies between findings. For example, some studies included first-episode unmedicated patients (48, 49), while others included chronic medicated patients (28, 30). However, the confounding effect of antipsychotics and disease chronicity remains controversial. One study found no significant difference in microstate parameters between chronic patients and first-episode patients with schizophrenia (27), while another study found different results (50). Furthermore, this difference may derive from the heterogeneity between samples. Because LLS is a highly heterogeneous disease, our sample size may not be large enough to cover the whole spectrum of LLS.

Microstate analysis showed an increased mean duration and decreased mean occurrence in patients with LLS compared with NCs. The duration of all classes also has a significant increase or a tendency to increase in patients with LLS. Similarly, significant decreases or a tendency to decrease of occurrence can be found in all classes of patients with LLS. This pattern of microstates is rather general and not specific to a certain class. Microstate patterns can reflect brain dynamics, capturing neural activity at the millisecond timescale (51). A less frequent and more prolonged microstate pattern suggested a slowing and inflexible brain dynamic in patients with LLS. Reduced brain dynamic was found to be related to poor cognitive functions (52, 53). Aging is also accompanied by a decline in brain dynamics (54, 55). Similar slowing microstate dynamics have been found in Lewy body dementia, which is viewed as underlying cognitive fluctuation and slowing information processing in Lewy body dementia (21). Therefore, the slowing microstate dynamics we found may reflect the widely reported marked cognitive impairments in LLS (4, 5).

In recent times, the link between EEG microstates and intrinsic neural oscillation has been elucidated. The formation and temporal dynamics of the microstates were proved to be dominated by the alpha-band rhythm (56–58). Among the four canonical microstate classes, class C has the strongest alpha oscillations over a wide cortex range (56). Alpha-band oscillations are considered to have an inhibitory function and play a crucial role in cognitive processes such as attention (59). Class C was also found to be more prominent in the resting state than in the task performance state (60). Therefore, it can be inferred that activation of class C may reflect inhibition of specific cortical areas and be involved in complex cognitive activities. Regular microstate class C characteristics represent organized inhibitory activity, essential for various cognitive functions. Furthermore, deviant class C parameters may represent a disruption of this order and, therefore, may lead to cognitive impairment. The current study found a significant decrease in the occurrence of class C in LLS, and the less frequent class C may contribute to disorganized brain function, thereby leading to the cognitive and psychotic symptoms of LLS.

Microstate syntax analysis found altered microstate transition probabilities in LLS. Several studies have reported the presence of abnormal microstate syntax in patients with schizophrenia; however, the results are somewhat inconsistent (23, 33, 49). It has been suggested that microstate syntax may represent the sequential activation of distinct brain networks. Therefore, the abnormal microstate syntax in LLS may represent a disorganized operation of brain network switching, giving rise to the aberrant behaviors in LLS. Based on the resting-state networks correlated to the four microstates (17), increased transition probabilities between classes B and D in LLS can be inferred as an enhanced interaction between those key nodes located in visual and attention networks. Conversely, decreased transition probabilities from classes A to B and from classes A to C may reflect decreased interaction between key nodes in the corresponding networks.

We found increased global omega complexity and anterior omega complexity in LLS compared with NCs, whereas no significant difference was found for omega complexity in the posterior region between groups. Omega complexity is considered a measure of synchronization of the distributed electrical activities or the number of independent neural processes (35). Increased complexity reflects reduced cooperation and enhanced independence of spatially distributed brain activity, and vice versa. Higher complexity can be found in relatively active states, thereby suggesting an increase in the independent information processing process. For example, omega complexity increases in eyes-open conditions and decreases during sleep (61, 62). To our knowledge, three studies investigated omega complexity in patients with schizophrenia. Irisawa et al. found increased global omega complexity in patients with schizophrenia (42). Saito et al. found that anterior omega complexity is significantly increased in schizophrenia (40). Kikuchi et al. revealed that schizophrenic patients have higher omega complexity in gamma and below-gamma bands, and the frontal area contributes significantly to the higher omega complexity (41). This accumulated evidence indicates decreased cooperativity and loosened connectivity of the active neural processes of schizophrenia, particularly in the anterior area, in line with the hypothesis that symptoms of schizophrenia are caused by disconnected brain networks, including those located in the anterior area (46, 63, 64). In line with previous studies on schizophrenia in younger people, we found significantly higher omega complexity of the whole brain and anterior region in LLS compared with NCs, suggesting that LLS has loosened connectivity of brain networks, especially those located in the anterior area.

This study has several limitations that should be considered when interpreting the findings. First, assessments of clinical symptoms and cognitive function were not performed. Further studies with detailed clinical and neuropsychological assessments are needed to investigate the relationship between schizophrenic symptoms, cognitive function, and electrophysiological indicators in LLS patients. Second, the confounding factors of drugs and comorbidities were not controlled. All participants in this study were older people. Some of them may have complex health problems (such as hypertension, diabetes, and coronary heart disease) and take various medications, potentially exhibiting confounding effects on brain function. Future studies could focus specifically on this potential effect. Third, considering the highly heterogeneous nature of schizophrenia spectrum disorders, the sample size of this study was small. Future studies with larger sample sizes are needed to confirm the findings.

In conclusion, this study revealed altered EEG microstate parameters, microstate syntax, and omega complexity in patients with LLS, reflecting disturbed brain networks underlying the symptoms of LLS. Our findings provide a better understanding of the pathophysiology of LLS and may facilitate the clinical application of EEG microstate and omega complexity. Because microstate was found to be an indicator for TMS efficacy (32), and microstate-based neurofeedback may serve as a therapy for schizophrenia patients (31), clarifying the microstate pattern of LLS may facilitate treatment strategies and the identification of intervention targets for LLS.

## Data Availability Statement

The raw data supporting the conclusions of this article will be made available by the authors, without undue reservation.

## Ethics Statement

The studies involving human participants were reviewed and approved by the Ethics Committees of the Affiliated Brain Hospital of Guangzhou Medical University. The patients/participants provided their written informed consent to participate in this study.

## Author Contributions

All authors contributed to the writing and revision of the manuscript, read, and approved the final manuscript.

## Funding

This study was supported by the National Natural Science Foundation of China (No. 82171533), Foundation of Guangdong Province, China (2022A1515011623), Medical Scientific Technology Research Foundation of Guangdong Province, China, (No. A2020446), Key Medical Specialty Construction Project of Traditional Chinese Medical Science in the 13th Five-Year Plan of Guangdong Province, and Key Medical Specialty Construction Project of Traditional Chinese Medical Science of Guangzhou (2020–2022). The funders had no role in the study design, data collection and analysis, decision to publish or preparation of the manuscript.

## Conflict of Interest

The authors declare that the research was conducted in the absence of any commercial or financial relationships that could be construed as a potential conflict of interest.

## Publisher's Note

All claims expressed in this article are solely those of the authors and do not necessarily represent those of their affiliated organizations, or those of the publisher, the editors and the reviewers. Any product that may be evaluated in this article, or claim that may be made by its manufacturer, is not guaranteed or endorsed by the publisher.
