# Supplementary material for: Altered Microstate Dynamics and Spatial Complexity in Late-Life Schizophrenia
Source: Front Psychiatry. 2022 Jun 27;13:907802. doi: 10.3389/fpsyt.2022.907802 (PMC9271628; doi:10.3389/fpsyt.2022.907802)
Supplement: Supplementary file 1 [file Table_1.DOCX]

**Supplementary material**

**Figure S1. The flowchart of EEG preprocessing**


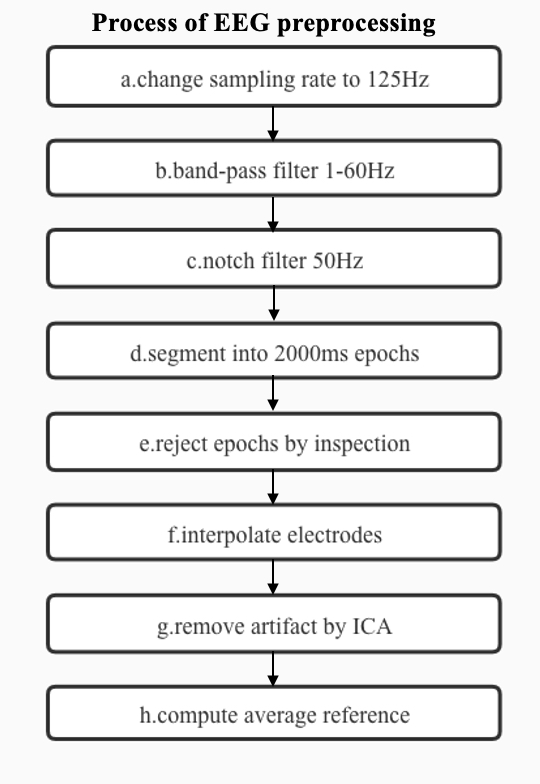


**Figure S2. The flowchart of omega complexity analysis**


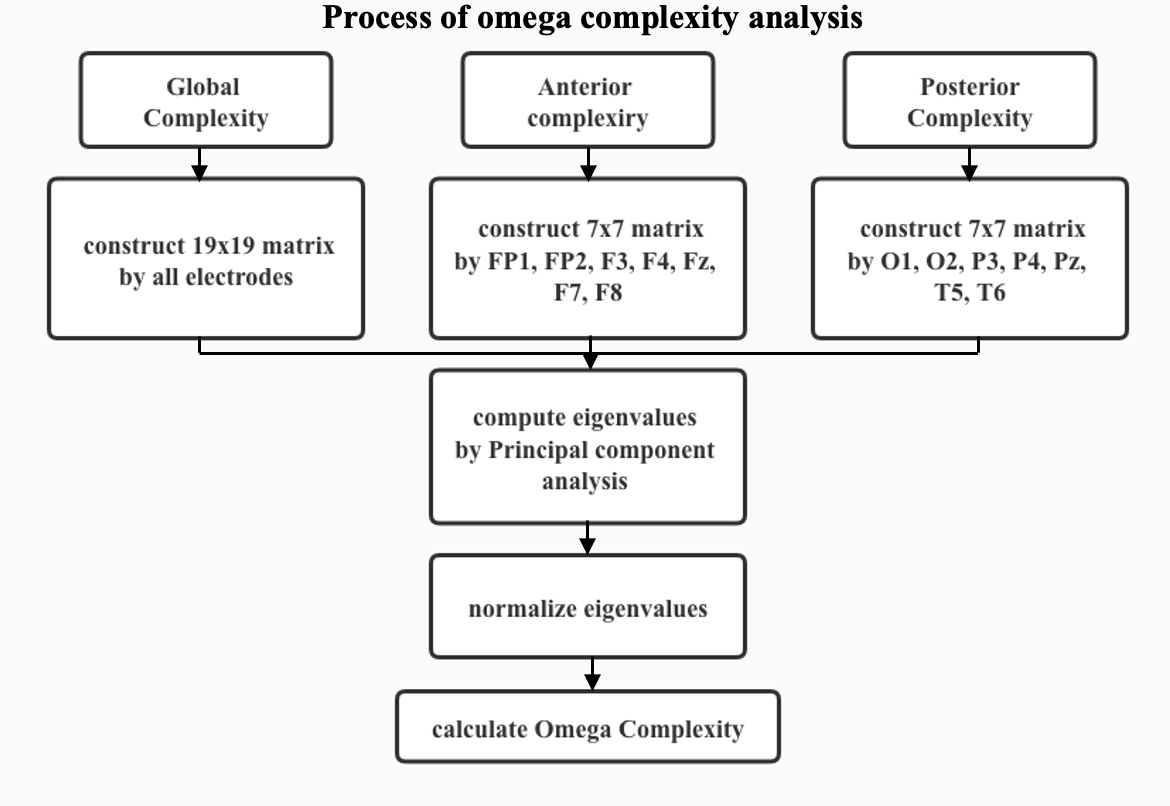


**Table S1. Microstate parameters and results of statistical test (post-hoc univariate ANCOVA) between LLS and NCs**

|  | LLS | NCs | F | *P* | η2 |
| --- | --- | --- | --- | --- | --- |
| Duration (ms) |  |  |  |  |  |
| Class A | 60.3±16.5 | 52.9±8.1 | 6.656 | **0.012** | 0.013 |
| Class B | 64.4±15.8 | 59.8±10.3 | 2.665 | 0.107 | 0.034 |
| Class C | 68.9±25.7 | 62.5±15.9 | 1.764 | 0.188 | 0.023 |
| Class D | 66.2±13.6 | 50.9±14.5 | 22.589 | **<0.001** | 0.229 |
| Occurrence |  |  |  |  |  |
| Class A | 3.54±1.15 | 4.94±1.06 | 30.884 | **<0.001** | 0.289 |
| Class B | 3.87±0.99 | 4.60±1.00 | 9.873 | **0.002** | 0.115 |
| Class C | 4.31±1.06 | 4.81±1.09 | 4.217 | **0.043** | 0.053 |
| Class D | 3.75±1.01 | 3.81±1.13 | 0.113 | 0.737 | 0.001 |
| Duration (%) |  |  |  |  |  |
| Class A | 21.39±8.72 | 25.21±6.08 | 5.031 | **0.028** | 0.062 |
| Class B | 24.40±6.38 | 26.45±6.06 | 1.904 | 0.172 | 0.024 |
| Class C | 29.28±11.44 | 29.10±9.60 | 0.009 | 0.924 | <0.001 |
| Class D | 24.92±8.57 | 19.23±8.24 | 8.489 | **0.005** | 0.100 |

Parameters were show as mean ± s.d.

LLS = late-life schizophrenia

NCs = normal controls

Significant differences (*P* ≤ 0.05) were showed as bold value.
